# Renaissance minds in 21st century science

**DOI:** 10.1186/s13059-020-01985-6

**Published:** 2020-03-13

**Authors:** Itai Yanai, Martin Lercher

**Affiliations:** 1 Institute for Computational Medicine, NYU Langone Health, New York, NY 10016 USA; 2 Institute for Computer Science & Department of Biology, Heinrich Heine University, 40225 Düsseldorf, Germany

The hypothesis-testing mode of science, which François Jacob called “day science,” operates within the confines of a particular scientific field. As highly specialized experts, we confidently and safely follow the protocols of our paradigms and research programs [1, 2]. But there is another side of science, which Jacob called “night science”: the much less structured process by which new ideas arise and questions and hypotheses are generated [3, 4]. While day science is compartmentalized, night science is truly interdisciplinary. You may bring an answer from your home field to another discipline, or conversely, venturing into another field may let you discover a route towards answering a research question in your main discipline. To be most creative, we may be best off cultivating interests in many areas, much like Renaissance thinkers such as Leonardo da Vinci or Galileo Galilei. But this creativity-enhancing interdisciplinarity comes at a price we may call “expert’s dilemma”: with your loss of status as a highly focused expert comes a loss of credibility, making it harder to get your work accepted by your peers. To resolve the dilemma, we must find our own balance between disciplinary day science expertise and interdisciplinary night science creativity.

## Bringing it all back home

Only 3 years after bursting onto the scene, Bob Dylan was already a folk music legend. His protest songs were the soundtrack of the anti-war movement that questioned America’s military engagement in Vietnam. Dylan’s lyrics, in particular, drew people in; after all, you do not get a Nobel Prize in literature for your music [5]. But then, in March 1965, Dylan set off a controversy with his fifth album, “Bringing it all back home.” The album had one side with more instant classic folk songs, such as “Mr. Tambourine Man.” But on the other side of the album, Dylan jumped right out of the folk genre. While before, his singing was only accompanied by an acoustic guitar and harmonica, these were rock songs, framed by drums, electric guitars, and electric bass. This “plugging in” was met by controversy from his fans and critics. Many of his biggest fans in the folk crowd hated it.

Modern science is probably even more categorically structured than music, with disciplines instead of genres. Similarly to how many folk music fans felt that Dylan should have remained in folk, scientists are generally expected to focus their career on a subfield of their discipline. If you work on genome biology, your group is likely a subdivision of the Department of Biology within the School of Natural Sciences. Such restricted playing fields will govern almost everything in our professional lives: the specialized conferences we attend, the topical journals where we publish, the study sections we select within our funding agency, the courses we teach, and the departments to which we get hired [6].

There is a good case to be made for why closely knit disciplines promote scientific progress. First of all, its structure makes day science more efficient. A community sharing a common established knowledge can move ahead to new knowledge. When your paper is reviewed for a journal or presented at a conference, it helps if you can rely on a core set of established ideas, so you can focus on the new aspects. But if the benefits of structured disciplines make you think that the next big idea in your field will come only from inside the field, you might be wrong.

## Night science is interdisciplinary

For Bob Dylan, the feel of a particular genre—be it country, rock, or blues—served to inspire his ideas that were searching for expression beyond boundaries. It was the recklessness and volatility of rock that allowed him to express the grudging anthem of “Like a Rolling Stone,” and it was the country medium that enabled “Lay Lady Lay.” The boundaries of a specific genre would have restricted the reach of Dylan’s songwriting. Arguably, Dylan writes and performs his best work precisely because he is able to transcend the constraints of particular musical styles. Dylan, then, is a prime example of a “Renaissance mind,” but the phenomenon is general: music has genres, but the musicians themselves may be most creative when they explore the full realm of possibilities within their reach.

Similarly, the borders between scientific fields and disciplines are not natural boundaries; really, there are no boundaries. Disciplines, fields, and subfields are just one way of clustering knowledge and methodology on increasingly fine-grained levels, but this clustering is not unique, and there is not even an obvious optimality criterion for the clusters. Many boundaries may simply reflect the way in which a field developed historically. Working within the confines of a field may help us to structure insights and ideas, but—similar to a musician’s fixation on a certain genre—the boundaries can impede our creativity and restrain our advances into certain directions. During our most creative night science moments, when we come up with potential solutions for problems and dream up hypotheses, when we need to make new and unexpected connections, we are better off if our mind is free to transcend the fields and disciplines. After all, if there were no boxes, we would not have to think outside of them. This kind of thinking may also be called horizontal [7] or lateral thinking [8].

To transgress the boundaries of a field, it is highly useful to have an understanding of multiple disciplines, either as a person or as a team, as this provides more opportunities to make connections. In the modern practice of science, the interdisciplinary aspect is often interpreted as a collaboration between scientists that work side by side in different disciplines. But true interdisciplinarity—even in a collaborative framework—requires us to *think* across fields. At some point, someone on the team will need to have that idea, and that someone will likely be the one with access to multiple fields. Thus, while the framework of science is disciplinary, a scientist’s creativity benefits from interdisciplinarity. This may explain why so many eminent biologists were originally educated in a different field: just think of Max Delbrück, Mary-Claire King, or Francis Crick. But there is also an important role for large and diverse teams: if more varied ways of thinking, more diverse ideas come together at the water fountain, they provide a fertile ground for making connections across borders—the modern workplace replacement of the traditional café, where creative people have traditionally met to exchange ideas [9].

A sizable minority of scientists feel comfortable away from their original field of expertise. They may specialize in a certain approach and be drawn to a new field because it generates exciting new data on which their approach can be applied, or they may first touch a foreign field as a side aspect in one of their research projects and then feel drawn into it. Many such scientists become “nomads,” switching fields every few years along their career. One true Renaissance mind, who frequently jumped between fields of mathematics, was Paul Erdös. Legend has it that for most of his life, he traveled from collaborator to collaborator, staying at the collaborator’s house until the work was through, and then asking with whom he should work next [10]. Together with each new collaborator, he would identify the problem that most interested them both. He had co-authored manuscripts with so many other mathematicians by the end of his life that it became fashionable to state your “Erdös number”—the degrees of separation that you have from him according to co-authorships (the scientific equivalent of the “six degrees of Kevin Bacon,” and likely its origin). Erdös had a catch phrase he would say as his next collaborator first opened the door, a beautiful summary of the Renaissance mind’s attitude: “My brain is open.”

## Expert’s dilemma

Does increased interdisciplinarity really lead to more insights? Meta-research—research on research—has investigated this using bibliometric approaches: a paper’s impact can be approximated by the number of citations it receives, while its interdisciplinarity is reflected in the diversity of the works it cites. The results are contradictory. Some studies found that adding research fields to a work was associated with an increase in impact, while other studies found that more interdisciplinarity is not necessarily better [11–16]. Digging deeper into the association between impact and interdisciplinarity has revealed its benefits, but also its costs. Of particular utility is the distinction between different forms of interdisciplinarity. A positive correlation was detected between impact and the variance of the references’ fields; however, if the different fields in the references were too balanced or if the fields were too distant from each other, this was associated with a lower number of citations instead [15]. Night science explorations may thus be most fruitful if they explore the adjacent possible [9]—the undiscovered knowledge that is still within reach from a given discipline, even if we have to stretch out beyond the field’s artificial borders. Viewing these relationships from the angle of the individual scientists, though, unearths an uneasy truth: those who engage in interdisciplinary research tend to be less productive than the experts [17]. At least in part, this may be a reflection of the difficulties involved in publishing interdisciplinary work.

**Fig. 1:**
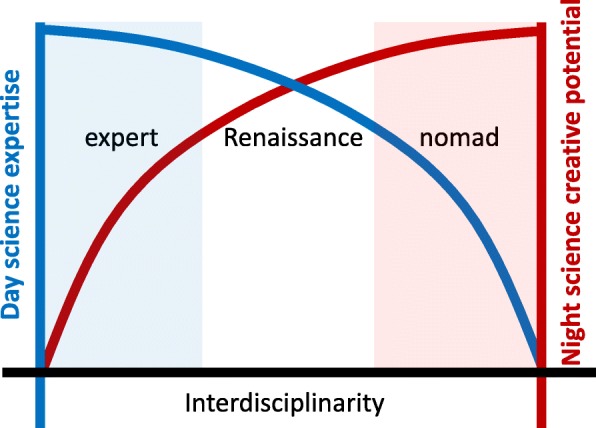
Expert’s dilemma. An increase in a scientist’s abilities for creative interdisciplinary thinking is almost inevitably linked to a loss in expertise and thus credibility in their home field, reducing the acceptability of the work to their peers

Such meta-research has quantified what many of us have experienced first-hand: a tension between the disciplinary and interdisciplinary aspects of science. As we know more about other fields, our creativity can venture further and in other directions. This empowers our night science. But on the other hand, an interdisciplinary scientist will likely lack some of the intimate knowledge expected of experts in his or her main field, and many peers will see this as an indication that this person is not to be trusted. There are a few polymaths, true experts in multiple fields, but for most of us, substantial knowledge about a second field can only be acquired at the cost of reduced expertise in our primary field. This is what we call “expert’s dilemma”: the more interdisciplinary you become, the less credibility you may have with your peers (Fig. 1). Thus, while the interdisciplinary person coming in may have a good idea, it might be dismissed because he or she does not know the ins and outs of the field, and when it is time for a grant panel or a journal editor to evaluate the work, why take it seriously? So while scientists sing the praises of interdisciplinarity, actually working in an interdisciplinary manner as a person can be a professional liability. Interdisciplinarity certainly enhances our night science creativity, but it may at the same time stifle our day time careers.

In response to the expert’s dilemma, different scientists have settled on different positions along the spectrum. Some scientists feel comfortable working at the left extreme of the plot, as a highly specialized expert in a particular field. They will spend many years, often decades, to arrive at an intimate understanding of a particular system. The discovery of the molecular pathway of programmed cell death [18] and protein degradation [19] is an example of this strategy. And of course, there are always the Erdös’s of the world who are continuously changing fields—true scientific nomads. But as always, a middle ground may provide the best tradeoff and may constitute the answer to expert’s dilemma.

## The idea import/export business

Interdisciplinary creativity is a two-way street. You may realize that a concept or a methodology, or maybe even just an analogy, from another field can aid in developing an answer to a question in your home field. Conversely, a concept or methodology from your home field may help in answering an open question in another field; the connection you discover might even lead to a new question in that field. Table 1 lists several examples in both directions. In all of these examples, the link discovered between the fields was not obvious and could not have been made by thinking purely within a single discipline. Rather, the creative act of finding the link was made possible by the scientist’s interdisciplinary thinking.

**Table 1:** Examples of idea importing and exporting across scientific fields

Importing an idea from another field	Exporting an idea to another field
Cancer: bringing in evolutionary thinking to explain the process [20] (see text).	Network thinking: applying network analysis methods to Internet robustness [21] (see text).
Gene regulation: bringing in concepts from electrical engineering to analyze gene regulatory networks [22, 23].	Quantum computer: bringing quantum physics to computer science [24].
DNA sequence analysis: using dynamic programming to align sequences [25].	CRISPR/Cas: bringing evolution and immunology to genome editing [26, 27].
P granules: modeling P granules as having liquid-like properties [28].	Ancient DNA: applying genomics to unravel ancient human history [24, 29].
Interactions among yeast cells: using game theory from economics (prisoner’s dilemma) to explain cellular interactions [30, 31].	Memes: applying evolution to model cultural changes [32].

A classic example of importing an idea from another field is the application of the theory of natural selection to the field of cancer. Wilhelm Roux, a German zoologist born in 1850, is best known for his pioneering work in experimental embryology and for the establishment of the first tissue culture. But aside from his focal work on embryology, Roux was fascinated by Charles Darwin’s books on the role of natural selection in the evolution of species. In what can only be considered a great night science moment, it dawned on Roux that natural selection was such a general principle that it should also apply to competition between cells inside the body. Roux published his ideas in his 1881 book, *The struggle of parts in the organism*; much of his later day science was devoted to testing the general ideas first laid out in the book [33]. It took the mainstream of cancer research over a hundred years to absorb this idea, but as we move further into the twenty-first century, few cancer researchers doubt that the spread of cancer cells is governed by an interplay between mutation and selection. The principles of natural selection have also been applied outside of biology. As early as 1873, Harper’s New Monthly Magazine wrote: “By the principle which Darwin describes as natural selection, short words are gaining the advantage over long words, [...] and local idioms are everywhere in disadvantage”—the origin of the idea of memes [32], ideas that spread by manipulating human brains.

Once an idea is generated in one field, it may prove so widely applicable as to lead people from that discipline to insights in disparate fields—see the right side of Table 1. A good example is the application of a suite of network analyses across disciplines, led by Albert-László Barabási. In 1999, Barabási and his then graduate student Réka Albert reported that many networks—the Internet, the citation patterns in science, or the collaboration graph of movie actors—have a peculiar property in common. These networks are “scale free”: at any level of magnification, they contain a few exceedingly popular nodes with many connections, while most other nodes only have a single connection [34]. Searching to fund this work, Barabási explored different calls far beyond the fields he had ever worked in. In his book *Linked* [21], he describes coming across calls from the DARPA agency on “technologies that will allow the computer networks of the future to be resistant to attacks and continue to provide network services.” The connection between this call and his work was tenuous at best, but applying their approaches to the topic of robustness, Barabási’s team of outsiders to the field of Internet security had an important insight: such networks are very robust to error, yet remarkably vulnerable to attack [35].

## Expert by day, Renaissance mind by night

How can we increase our night science creativity by thinking across disciplines? The first step is simply to be aware of the distinctions between day and night science, and between disciplines and scientists. Disciplines are structured frameworks that guide out day science, but that we may have to traverse in our creative periods. Night science is about discovering the unknown and the unexpected, and thus, there can be no established maps to follow. To expand our interdisciplinary minds, it may be a good idea to read about many things—and doing this in a superficial way is nothing to be ashamed of; on the contrary, it may often be a necessity. While we cannot become an expert on everything, cultivating wide interests—for example, through popular science books—provides a rough map of the questions that people in other fields are wondering about, the methodologies they use, and the concepts and analogies that guide their thinking. Attending talks that have only a marginal overlap with your work can also be inspiring. By connecting their data and their questions to your own expertise, you may come up with new ideas: a way to use their data to shed light on a question in your field, or a way to address their questions with methodologies familiar to you. This way, you will still be able to skillfully navigate your particular field, while at the same time you gain a familiarity with other fields for inspiration: think like an expert by day, but with a Renaissance mind by night.

Experts will remain crucial to scientific progress, and universities, which developed an intricate disciplinary structure for good reasons. Specialists rule by day. But scientific creativity—night science—is enhanced by our ability to move between disciplines. The pendulum swings back and forth over the years on the emphasis of interdisciplinarity in science. Even when it is called for, interdisciplinarity is often seen from a purely day science perspective, emphasizing the role of large teams of diverse but highly focused, disciplinary experts [36]. In contrast, interdisciplinary creativity seems to thrive with “teams” of only one or two scientists, and often occurs when a scientist without formal training in a given field ventures into it in night science excursions (and, in many cases, subsequently even during day science). So if you always wanted to explore other fields, but felt it might be a waste of your time: "don't think twice, it's alright".
